# Wearable Edge AI Applications for Ecological Environments

**DOI:** 10.3390/s21155082

**Published:** 2021-07-27

**Authors:** Mateus C. Silva, Jonathan C. F. da Silva, Saul Delabrida, Andrea G. C. Bianchi, Sérvio P. Ribeiro, Jorge Sá Silva, Ricardo A. R. Oliveira

**Affiliations:** 1 Computer Science Department, Federal University of Ouro Preto, Ouro Preto 35400-000, Brazil; jonathan.cristavao@aluno.ufop.edu.br (J.C.F.d.S.); saul.delabrida@ufop.edu.br (S.D.); andrea@ufop.edu.br (A.G.C.B.); rabelo@ufop.edu.br (R.A.R.O.); 2 Biology Department, Federal University of Ouro Preto, Ouro Preto 35400-000, Brazil; spribeiro@ufop.edu.br; 3 Department of Electrical and Computer Engineering, INESC Coimbra, University of Coimbra, P-3030 Coimbra, Portugal; sasilva@deec.uc.pt

**Keywords:** (multipurpose) wearable edge AI, edge computing, wearable computing, computer vision, embedded systems

## Abstract

Ecological environments research helps to assess the impacts on forests and managing forests. The usage of novel software and hardware technologies enforces the solution of tasks related to this problem. In addition, the lack of connectivity for large data throughput raises the demand for edge-computing-based solutions towards this goal. Therefore, in this work, we evaluate the opportunity of using a Wearable edge AI concept in a forest environment. For this matter, we propose a new approach to the hardware/software co-design process. We also address the possibility of creating wearable edge AI, where the wireless personal and body area networks are platforms for building applications using edge AI. Finally, we evaluate a case study to test the possibility of performing an edge AI task in a wearable-based environment. Thus, in this work, we evaluate the system to achieve the desired task, the hardware resource and performance, and the network latency associated with each part of the process. Through this work, we validated both the design pattern review and case study. In the case study, the developed algorithms could classify diseased leaves with a circa 90% accuracy with the proposed technique in the field. This results can be reviewed in the laboratory with more modern models that reached up to 96% global accuracy. The system could also perform the desired tasks with a quality factor of 0.95, considering the usage of three devices. Finally, it detected a disease epicenter with an offset of circa 0.5 m in a 6 m × 6 m × 12 m space. These results enforce the usage of the proposed methods in the targeted environment and the proposed changes in the co-design pattern.

## 1. Introduction

The application of multipurpose hardware and innovative software technologies helps to provide new services in various environments [1]. Networking, operation, and management are some of the main aspects of future network-based systems. The growth of devices with networking capabilities and hardware miniaturization empower the development of the internet of things (IoT) [2].

Within the context of operation and management features in network-based environments, some of the principal technologies involved regard machine learning [1]. The evolution of the IoT concept also provides a means to bring applications from the cloud to the edge [3]. This perspective brings the processing, operation, and management closer to data acquisition, avoiding large data throughput, and latency. The fusion of edge devices into the IoT concept benefits several applications, such as smart cities, healthcare, and environmental monitoring [3].

Environmental monitoring has a crucial role in managing natural or agricultural environments [4]. These systems are also essential to assess climate change and its impacts. Manfreda et al. [4] enforce that most monitoring systems are either ground-based, airborne, satellite-based, or a combination of them. For ground-based measurements, researchers should consider the possibility of using multi-sensor- and IoT-based wearable systems [5,6,7]. The combined data can be used to perform three-dimensional inferences from the acquired information [8].

Leaf aspects are indicators of ecosystem health. Therefore, visual aspects that indicate herbivory, aging, and disease must be observed throughout ecological canopy studies. Bielczynski et al. [9] state that plant and leaf aging affects its performance on photosynthesis and photoprotective capability. In addition, disease detection is a relevant issue in both agricultural and ecological contexts [10,11,12]. Tree diseases are influenced by the whole ecosystem’s health, working as individual indicators of biotic, biophysical, and environmental stresses [13]. Hence, detecting and understanding these diseases help to assess environmental health.

### 1.1. Main Objectives and Contributions

In this work, we evaluate the aspects of creating wearable edge AI applications for studying ecological environments. For this matter, we performed two tasks, as the two main objectives of this text. At first, we propose a novel project pipeline approach, reviewing the hardware/software co-design pattern for creating robust IoT- and edge computing-based appliances. This novel approach considers that the architecture must be integrated and validated in parallel with developing the hardware and software traits. Then, we propose a case study based on validated research methods to apply machine learning (ML) tools in a wearable edge AI collaborative environment. We evaluate the constraints for this application considering hardware, software, and architectural analyses. Some specific contributions are:

- A novel co-design pattern considering architectural constraints;
- A new architecture for performing studies and analysis in field research;
- A method for integrating existing and validated solutions in adjustable IoT- and edge computing-based environments.
- An evaluation of a ML tool for detecting diseases in leaves.

### 1.2. Text Organization

Up to this point, we introduced the subject of this study and some theoretical background that supports the developed research. The remainder of this paper is organized as follows: In Section 2, we review the literature for related work considering the main aspects of this solution. Section 3 presents the case study for evaluating the usage of this architecture in a collaborative environment. In Section 4, we define the materials and methods we use to validate this approach. We define hardware, software, and architectural traits that must be evaluated. Section 5 displays the results we obtained from the proposed tests. Finally, we discuss our results and conclusions in Section 6.

## 2. Related Work

In the last section, we presented part of the theoretical background for this work, reviewing some aspects relevant to this context. Nonetheless, it is required to understand the importance of this and other proposed contributions within the area. Thus, in this section, we present the search results in the literature for related work. We chose to search for aspects within three main branches: Wearable computing in field and forest research, edge and wearable Computing, and wearable edge AI. As we display in the following subsection, these areas are new and promising, and this research works some gaps in the research field.

### 2.1. Wearable Computing in Field and Forest Research

Wearable computing devices are generally used for entertainment in sports [14], and in the medical field, ref. [14] for monitoring the health of its users. Although health human monitoring application is considered relevant for field application, in this paper, we claim for alternatives that wearable devices should be a service provider to field researchers and practitioners. To this end, few works found in the literature give this focus on their investigations.

The proposed device in [15,16] can be used for simultaneous image processing as a sensor station to provide information about the environment. An Intel Edison development board was integrated into the architecture. In another approach, a smart helmet is proposed as an alternative to climb trees for researchers to perform their tasks [17].

In [18], the authors propose an interactive wearable device based on a sound system to create the sense of users and remote soundscape, enabling them to feel in nature despite them being in the city. In the agriculture field, wearable devices have been used to collect information from the user or environment [19].

We attributed the low number of works focused on wearable application to the field research due to the most traditional IoT paradigms. As a result, system designers tend to propose solutions integrated to the cloud platform, independent of the chosen paradigm (cloud-based, edge-based, and fog-based). Indeed, these approaches restrict the application because the field research environment has several connection restrictions. The following sections describe information toward the maturation of edge AI wearable devices application in forests.

### 2.2. Edge and Wearable Computing

The IoT context brings on several devices with networking capabilities, producing data, and receiving data-based decisions and insights. The scalability, availability, communication throughput, and latency in integrating these applications to a processing service brings the need to process data closer to the edge [20]. Thus, the concept of edge computing emerges from this condition [21]. This trend is enforced when considering mobile [22] and wearable [23] computing.

Wearable computing specifically brings applications to improve the users’ cognition. The increasing number of applications towards this goal is bringing user-centered applications in healthcare [24] and activity recognition [25], and many other appliances. In this context, the usage of edge computing decreases the latency of this assistive technology when compared to traditional cloud appliances [23]. Furthermore, as wearable computers are usually resource-restrained, edge computing provides a way to bring more processing to integrate with multipurpose wearable devices [26].

### 2.3. Wearable Edge AI

Usually, field research environments do not have the necessary infrastructure to integrate edge wearable devices and computer systems. Wearable edge AI is an alternative concept for providing services required to ecological researchers and practitioners. Its concept aims to provide local services based on artificial intelligence applications. This means embedded devices can perform machine learning models to assist a human in the decision-making process in real-time.

The increasing interest in machine learning, deep learning, and other computational intelligence applications raised a relevant topic of discussion regarding how to bring these algorithms to the edge. According to Chen and Ran [27], the main challenges regarding the usage of machine and deep learning when considering this aspect are latency, scalability, and privacy. Chen and Ran, and also Wang et al. [28] state that typical uses for these technologies are computer vision (CV) and natural language processing (NLP). Wang et al. also enforce that some relevant features in these applications are cost, reliability, latency, and privacy.

An increasing number of wearable computing applications are using edge computing to provide insights based on machine learning. For instance, there are appliances in health monitoring [29,30,31], ergonomics [32], activity tracking [33], and so on. An important aspect is that most of these applications are user-centered. They focus on monitoring the users’ conditions but have low integration on the environment monitoring. Although there are many applications and common features, the authors still do not define wearable edge AI as a single topic. Thus, in this work, we begin to formalize the constraints and design patterns for such applications.

## 3. Case Study

**Figure 1:**
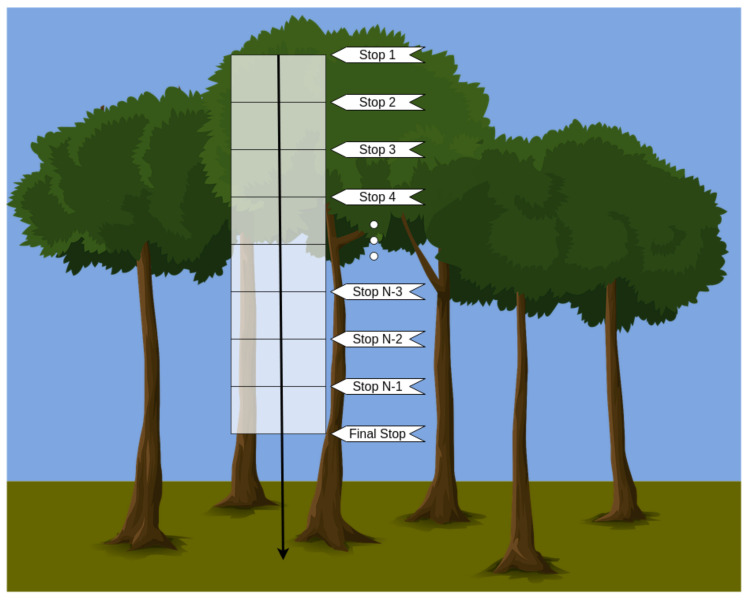
Illustration of the cylinder-transect study.

As we developed the edge AI system for leaf disease detection, the case study is a triangulation from three different climbers performing a cylinder-transect study, similarly to the one proposed by Ribeiro, Basset, and Kitching [34]. The researchers perform a downward climb within the canopy in this method, starting from the upper canopy. They sample leaves in horizontal transects, spaced with predetermined distance steps until they reach the final stop. In this first approach, we consider sampling using a background template to facilitate data segmentation. Usually, the last stop is at around 3 m from the floor. Figure 1 displays an illustration of the proposed method.

**Figure 2:**
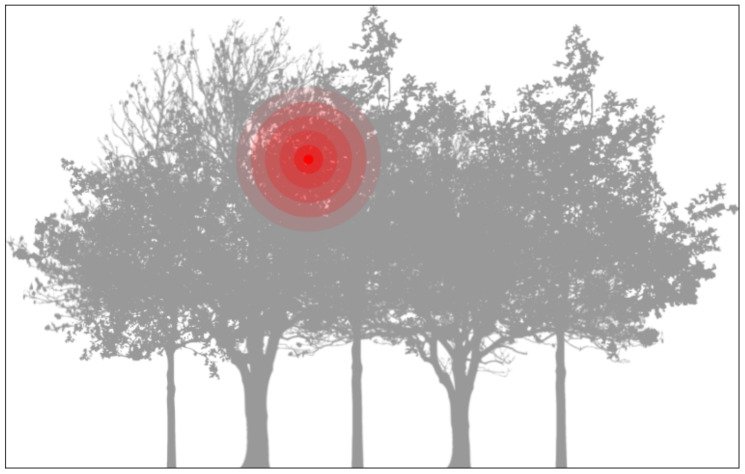
Example of a possible location for a disease spread. We model this spread using a spatially-distributed probability density function (PDF).

As stated, the leaf conditions are very relevant indicators of ecosystem health. For instance, García-Guzman et al. [35] displayed that in Mexican wet forests, the incidence of diseased leaves can be up to 65% in most infected areas, where it is circa 2% in low infected regions. With this baseline, we expect that this disease is distributed within higher and lower infection regions whenever a pathogen is present in a canopy. From this perspective, we model disease spread according to a probability density function (PDF), centered in the local with the highest infected percentage. Figure 2 displays an illustration of a density gradient based on a centered maximum.

We assume that this distribution is normal-shaped along with the canopy. Thus, the distribution can be modeled as a gaussian-based PDF geometric function. This function is presented in Equation (1). The advantage of this function is that the disease spread can be represented using only five parameters. The parameter $p_{0}$ represents the maximum incidence of the disease. As usual, the σ parameter represents the standard deviation. For simplifying purposes, we used a single standard deviation in all three spatial coordinates. The final three parameters compose the $\left(x_{0},y_{0},z_{0}\right)$ center of the distribution. The purpose of this paper is not to discuss the modeling process itself, but to provide a case study with simplicity and replicability:

(1) $$P\left(x,y,z\right)=p_{0}.e^{-\frac{{\left(x-x_{0}\right)}^{2}+{\left(y-y_{0}\right)}^{2}+{\left(z-z_{0}\right)}^{2}}{2\sigma }}.$$

The usage of Gaussian-based models to model disease spread is supported by some authors who previously worked with similar approaches. For instance, Soubeyrand, Enjalbert, and Sache [36] used Gaussian-based modeling to model the circular spread of airborne plant disease. Pokharel and Deardon [37] also performed modelings of infectious disease spread based on Gaussian distributions. Even in the COVID-19 context, Ketu and Mishra [38] also used Gaussian-based models to predict the disease distribution.

Although there are similar approaches in the literature, there are some key features from which we chose this modeling approach over Gaussian random processes (GRPs) or Gaussian process estimators (GPEs), as some of the authors. In the model proposed by Soubeyrand, Enjalbert, and Sache [36], the authors used GRPs to create a rough model based on circular functions over a two-dimensional space. As our objective in this paper is not to discuss disease modeling, we opted to create a smoother model based on a single spatial funcion. Pokharel and Deardon [37] propose the usage of Gaussian process approximations to create emulators (GPEs) of a dynamic disease spread model in two dimensions. The objective is somehow different, as the authors desire to model extra variables and processes that are not the object of this study.

Various researchers performing the cylinder-transect method in the canopy obtain the distribution of diseased and healthy leaves in well-known coordinates. Although the representation is simple, it is not trivial to perform a regression from density points in three-dimensional space to a continuous-space function. Thus, we propose using a heuristic method to obtain the parameters that better represent the original function.

## 4. Materials and Methods

In this section, we assess the present review of the HW/SW co-design principles into HW/SW/architecture co-design for developing edge computing-based solutions. The first stages are the requirements definition and general architecture proposal. Then, the design branches into architectural, software, and hardware traits for parallel implementation and validation. Finally, the architecture must be validated considering the case study. In this section, we present the proposals and tests to perform and validate each stage and branch.

### 4.1. Rethinking the Hardware/Software Co-Design for Edge AI Solutions

A significant pattern when designing novel solutions within embedded and wearable systems is the hardware/software co-design principle [39,40]. This directive guides the design and validation of parallel hardware and software aspects for the later integration on novel systems.

**Figure 3:**
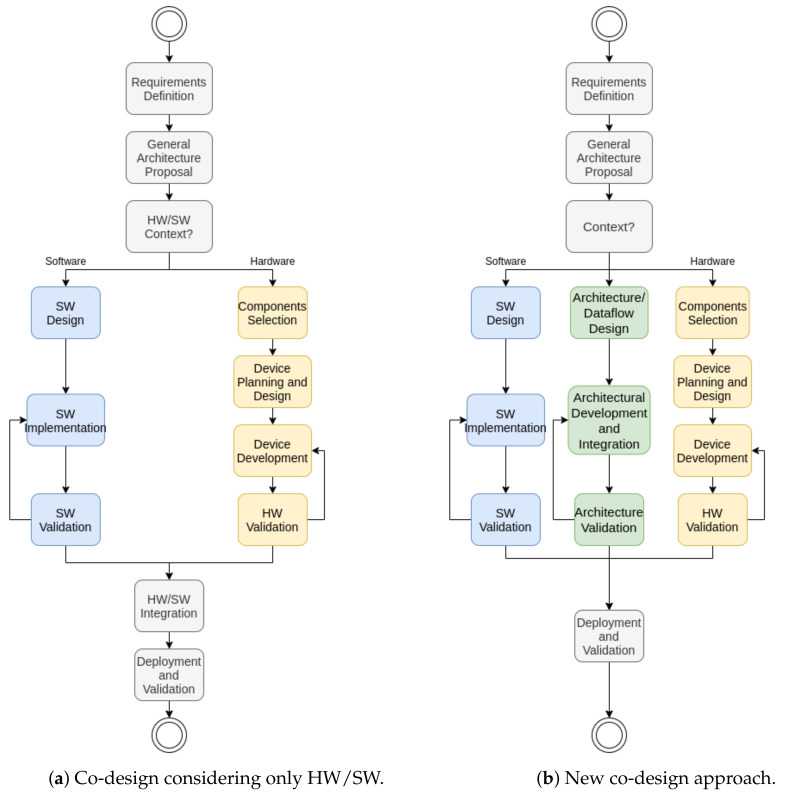
Co-design considering only HW/SW.

In this text, we propose a new approach to this pattern, considering that architectural factors must also be validated in parallel during the design process in edge computing and IoT approaches. Figure 3 displays the traditional and new diagrams for the co-design. Figure 3a displays the traditional approach for the hardware/software (HW/SW) co-design pattern. Figure 3b explores a new branch for designing and validating the architecture in parallel with hardware and software constraints.

In the traditional co-design approach, the process starts with a requirements definition and general architecture proposal. Then, the constraints are separated between hardware and software contexts. After that, the development of hardware and software aspects of the solution happens in parallel. Finally, the solution is only integrated after validating both aspects. This architecture is sound in high or low abstraction levels in developing single solutions considering both wearable and embedded systems. Nevertheless, this architecture is fragile when dealing with multiple and variable architectures. This weakness happens if the validation after the integration fails due to architectural traits. When thinking about a wearable edge AI, these factors must not be ignored.

Therefore, we propose that the architecture validation represents a new branch after context splitting. In the design process, the proposition of new devices must also identify the essential aspects of architectural design. They must also validate these designs while in the proposal process. Finally, as the integration happens in parallel, the last stage is the deployment and validation of all systems together. In the architecture branch, represented in green blocks in Figure 3b, there are three new stages:

- *Architecture/Dataflow Design*: In this stage, the proposal must identify how the devices communicate within the network. In the context of IoT and edge computing, devices communicate with each other providing services, insights, and information. Integrating devices in the same WBAN/WPAN, or even multiple devices with multiple WLAN users, requires a dataflow design.
- *Architectural Development and Integration*: After defining the roles of each device within the network, as well as the integration protocols, the architecture must be developed in parallel with the integration of hardware components and individual software traits.
- *Architecture Validation*: Like the other branches, the architecture must also be validated using formally-defined tests. This aspect enforces the design process and identifies flaws in the development process that must be assessed.

### 4.2. System Requirements

Wearable Systems are Embedded Systems. Therefore, the core requirements for every wearable system are the same as the general embedded systems requirements: Energy, robustness, timing, and communication [41]. They also have some further requirements due to their nature. They need to be comfortable and easy to use [42] and must augment reality through context-awareness [43].

In order to understand how these restraints apply to our desired context, we performed several interviews with the target users from the technology. From their knowledge, we estimated the energetic autonomy and robustness requirements in a qualitative or semi-quantitative form. Communication and synchronization requirements come from the proposed architecture features.

As this system needs to be taken into the field, it needs to work for hours without a battery recharge or replacement. It needs to be robust enough to take hits from branches and falling seeds or nuts. As we propose a distributed system in a WBAN-Environment, both systems need to communicate with an application, working as web server nodes. Finally, the communication needs to be efficient to stream the data through this local network.

### 4.3. General Architecture Proposal

**Figure 4:**
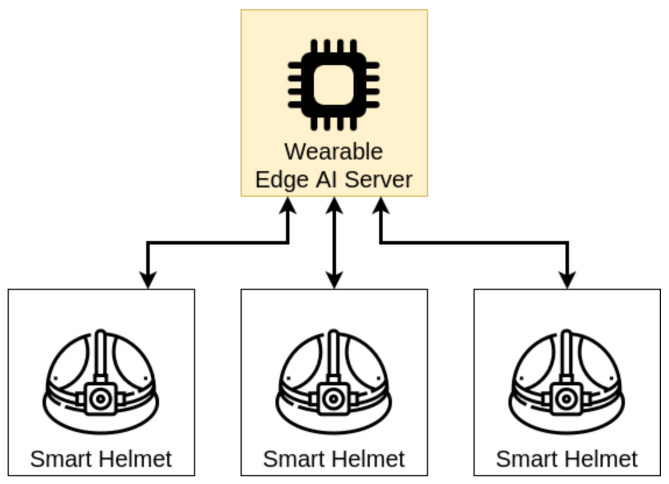
Proposed general architecture. The smart helmets use the wearable edge AI server to provide machine learning inferences.

The proposed solution works upon a wearable distributed system working in wireless body-area network (WBAN) and wireless local area networks (WLAN). This system is projected to allow information extraction through data fusion, image processing, and computer vision. Figure 4 presents the proposed architecture for this system.

The first aspect of this system is the physical core. As we propose a wearable system, it needs to be an unobtrusive system [44]. Therefore, we built the system over equipment that allows the user hands-free. The physical core which presents the best option for the wearable and research requirements is a helmet. In Silva et al. [45], we discussed some of the appliance constraints. Primarily, we estimated the energetic requirements and consumption for such a solution.

The following aspect is the construction of IoT-based sensor nodes. Each node is a computer-on-chip capable of reading single or multiple sensor data, pre-processing this data, and casting it over WBAN or WLAN. The chosen sensors to augment perception to the environment are a laser radar (LIDAR), a 9-degree-of-freedom inertial measurement unit (9DoF IMU), and a regular camera.

The computer-on-chip must be able to read the required I/O interfaces from each sensor. It must also be able to establish a wireless connection in the local body area. As wearable systems have strong energy constraints, the computer-on-chip must carry a low-power processor. Thus, ARM-based computer-on-chips are adequate solutions for this purpose.

In general, the candidate devices to perform as core applications for this system are ARM-based computer-on-chips, with multiple different I/O interfaces to attach the required sensors, and a network card capable of streaming the data through a local wireless connection.

We chose the Wi-Fi network standard (http://www.ieee802.org/11/, accessed on 22 July 2021) (IEEE 802.11) as WLAN/WBAN-interface. This choice was based on the ease of creating web server solutions, data throughput, and range for WBAN/WLAN. It was also based on the broader band capability to guarantee the connection quality, especially when dealing with camera streaming.

Within this network, each sensor node operates like a local webserver. Any application built should request the sensor data from each node over the WBAN/WLAN. The application performs a data fusion algorithm and augments reality.

### 4.4. Hardware Specification

From the proposal, there are two main hardware element decisions: The smart helmet hardware and the edge AI node hardware. For the smart helmet, we needed a modular and validated solution with an integrated camera. On the other hand, the edge AI node hardware selection stage must combine performance and portability.

#### 4.4.1. Smart Helmet Hardware

This project is a continuum evolution of a wearable device built for ecology environment use. Other papers were published by members of the research group, which had results previously discussed. The papers [17,45] describe the hardware specification and its evaluation. It is no focus of this paper to reproduce the evaluation previously discussed. Instead, we aim to integrate the proposed hardware on the edge-AI architecture in this scope.

This section provides a brief description of the wearable device built and provides the essential information and concepts to the proposed integration. The developed hardware is a helmet composed of sensors and a unit for data processing. It was built for researchers and professionals who use the climb tree technique to collect ecological environments. Section 3 describes more detail about their tasks and expectations.

**Figure 5:**
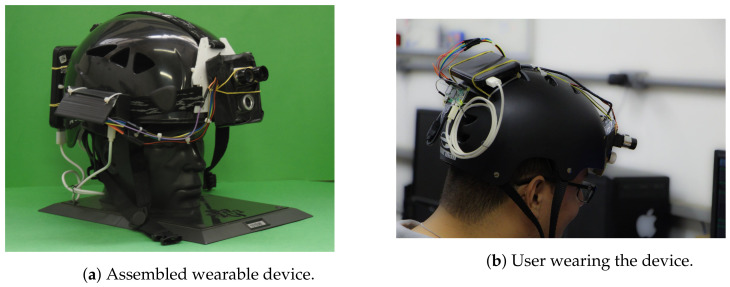
Assembled wearable device.

Figure 5 shows the wearable prototype. Figure 5a is a perspective of the front and side view of the device, and Figure 5b shows a user wearing the device from the back perspective. The hardware bears a LIDAR sensor connected to a unit processing for distance and 3D shape estimation of the sensing objects. A Raspberry Pi Zero W was adopted as unit data processing powered by a 5 V battery. We considered the wearable requirements and application requirements during the development process.

We used this prototype to perform individual tasks in this scope. As we have previously validated this solution’s sensing aspects, we mainly performed tests involving its computing capability in this work. As displayed in further tests, this prototype can perform some of the desired tasks but has limited capabilities when dealing with higher demand processing. Thus, this perspective justifies the proposed edge-based architecture. In the following section, we discuss the process of choosing edge computing hardware.

#### 4.4.2. Edge AI Server Node-Hardware Selection and Integration

Besides the smart helmet, another critical aspect of the general architecture is the edge AI server node. Thus, this hardware selection must consider embedded systems that are portable and may provide this stage with machine-to-machine communication. Our case study provides machine learning within the WBAN/WLAN context for creating a wearable edge AI perspective. In this text, we compare the performance of four hardware elements capable of providing this utility.

**Figure 6:**
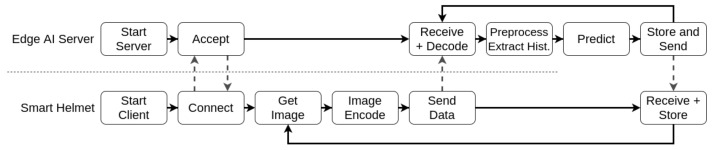
Edge AI service pipeline. In the proposed architecture, clients perform part of the processing, while the AI pipeline is provided by the edge AI server node.

At first, we created a pipeline, separating the elements processed locally and within the edge AI server node. Initially, the local systems acquire and encode the image, sending the encoded data to the edge server. In this server, the application loads a trained ML model and constantly receives encoded frames, decodes them, pre-processes and extracts the pseudospectrum, and evaluates it. The evaluation result is then stored by the edge AI server and sent back to the device for redundancy. Figure 6 displays the proposed pipeline for the edge AI server supporting a single client.

We considered various devices for creating the solution. We considered the Raspberry Pi Zero W, Raspberry Pi 3B, Raspberry Pi 3B+, and Jetson Nano platforms as suitable candidates for providing edge AI as a utility in the context of this wearable edge AI solution. All these solutions are commercial-off-the-shelf ARM-based computer-on-modules.

### 4.5. Edge AI Software

As presented in the introductory section, the conditions of leaves are essential indicators of the ecosystem’s health. Thus, we decided to evaluate the constraints of an edge AI element performing classifications of leaves within two classes: *Normal* and *diseased*. In this section, we cover the edge AI software applied in the case study.

**Figure 7:**
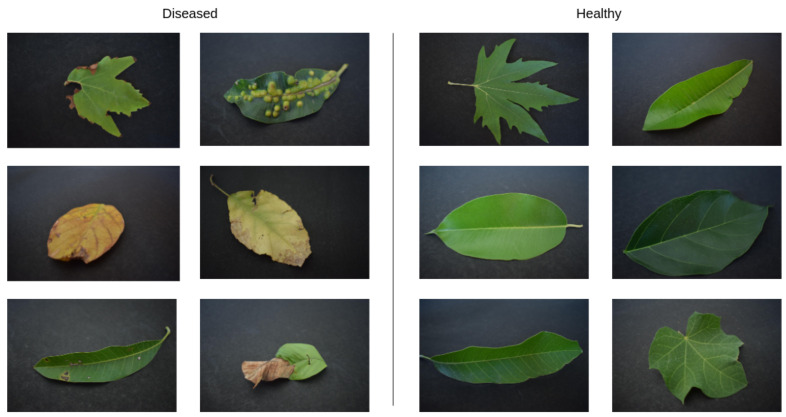
Sample of healthy and diseased leaf images obtained from the dataset.

The first step is to find a dataset with similar information to the one desired. After some search, we decided to apply the dataset from Chouhan, Kaul, and Singh [46]. They present a dataset with 4503 images of leaves with and without a disease. Within this set, 2278 images are from healthy leaves, and 2225 images are from diseased leaves. The leaves are from 12 different species. Figure 7 displays images of healthy and diseased images from the original dataset. In this database, the separation between diseased and healthy leaves is guaranteed by the creators. In all cases, any eventual difference in color and texture is caused only by disease. The images had a resolution of 6000 × 4000 pixels. To increase the speed of the test and use a resolution closer to most available cameras for embedded systems, we lowered the resolution to 900 × 600 pixels. The new resolution corresponds to 15% of the original size.

**Figure 8:**
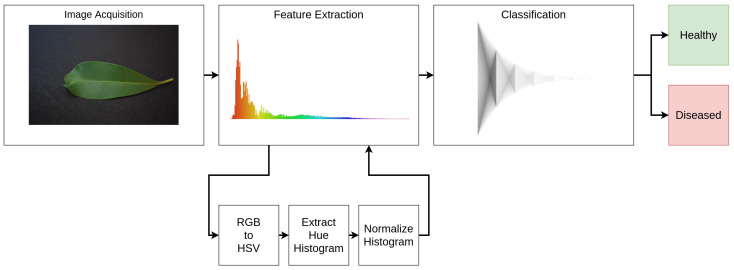
Data processing pipeline and associated substages. For the image extraction, the associated stages are the color space conversion and histogram extraction.

The process of extracting and classifying the data follows a classical pipeline. At first, we acquire the image and extract a feature vector. Then, we classify the image according to the features using an ML model, obtaining the binary classification result. Figure 8 displays the pipeline, with the substages associated with each main stage.

**Figure 9:**
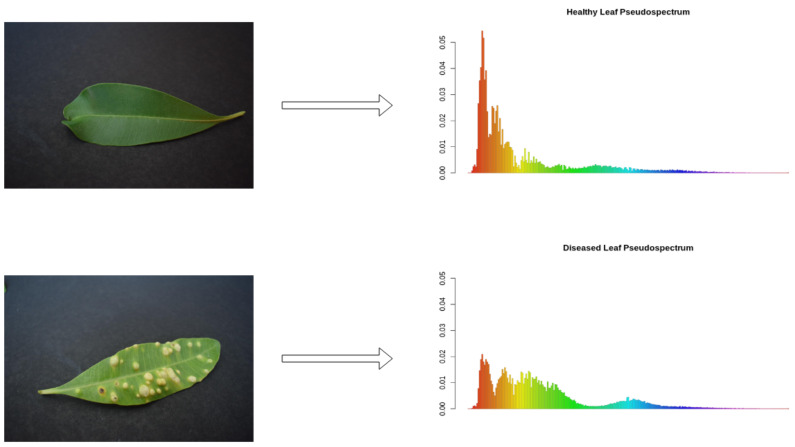
Pseudospectrum extraction samples.

In this context, we approach this problem by creating a pseudospectral analysis system [47]. In this pipeline, the feature vector is a pseudospectrum, corresponding to the extraction of the histogram from the Hue channel in the HSV color space. Finally, we divide the histogram by its sum, creating a probability density function (PDF) corresponding to the color distribution, henceforth named pseudospectrum. Figure 9 displays some examples of the pseudospectrum extraction.

**Figure 10:**
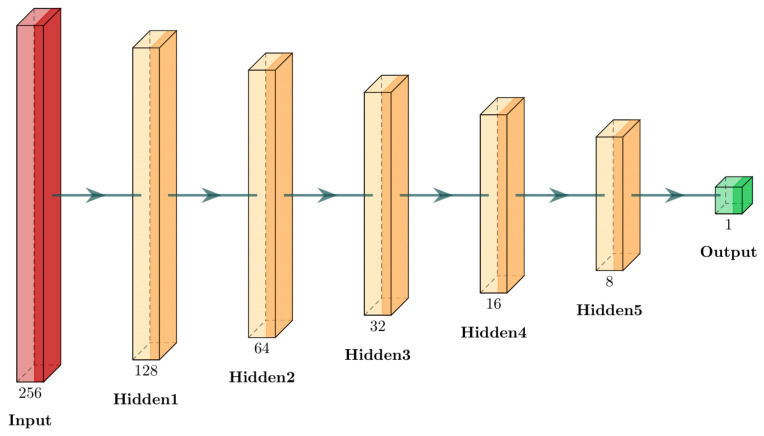
Neural network representation. The chosen model was a multi-layer perceptron (MLP). All layers are fully connected. The number beneath the blocks represents the number of neurons in each layer.

Then, we use a neural network to classify the leaf. For this matter, we used a traditional multi-layer perceptron (MLP) model to perform the calculations. Although the model is not new, we chose it because of its simplicity, and thus a better performance enhancement in an edge computing low-power hardware. Although the model is simple, it previously displayed interesting results when classifying citrus fruits [47]. In this context, we used a network taking 256 inputs from the pseudospectrum, with hidden layers containing 128, 64, 32, 16, and 8 neurons. The output was a binary classification of healthy or diseased. Figure 10 displays an overview of the network architecture.

**Figure 11:**
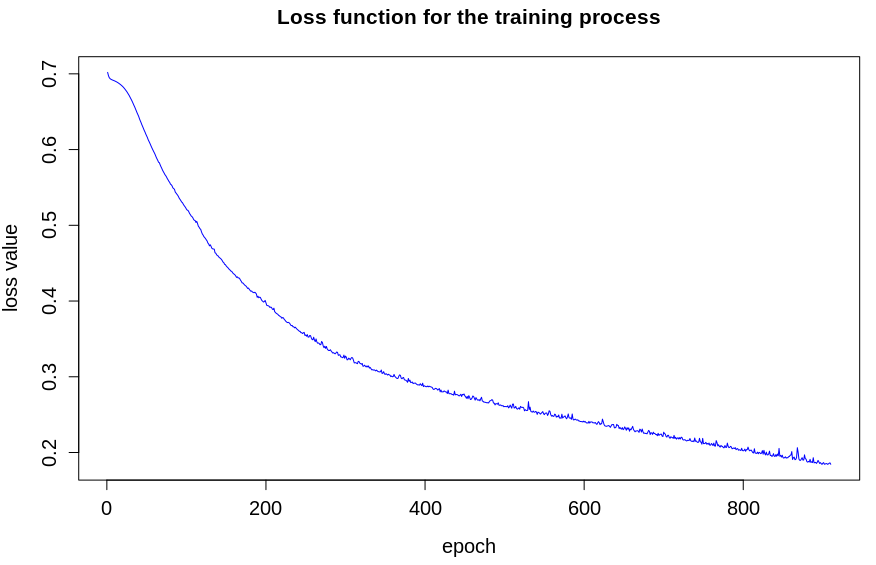
Loss function during the training process.

We used the *scikit-learn* framework to develop our model [48]. The training happened using a backpropagation algorithm, using a cross-entropy loss function. For training purposes, we separated the original images dataset into two subsets. In the first stage, we randomly selected 10% of the images of diseased and healthy leaves from each species to compose a test dataset. The remaining 90% composed a training set. We used 90% of the images from the training set to train the algorithm and the remainder 10% to validate. Figure 11 displays the behavior of the cross-entropy loss during the training. This training ends with an arbitrary convergence criterion, which is not improving the cross-entropy loss value of more than $10^{-5}$ for 10 consecutive epochs.

Besides this model, we also tested the perspective of a convolutional neural network (CNN) model to perform the predictions in the embedded hardware. This technique is more modern, but requires more computational power. Thus, we proposed a test that measured two aspects:

- How much improvement can a CNN obtain over a computer vision and MLP;
- How much performance the embedded system loses using this method over a traditional approach.

**Figure 12:**
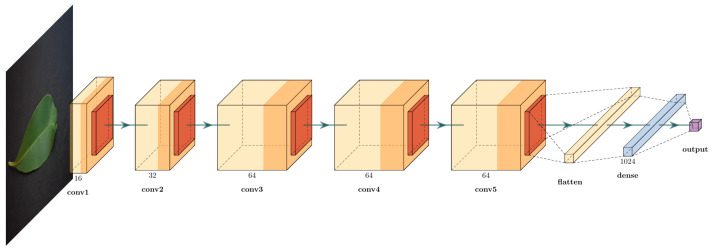
Proposed CNN model. The convolutional layers have 3 × 3 filters, with 2 × 2 pooling. The output is a single value obtained from a sigmoid activation function.

We created a simple CNN model that approaches this process. Figure 12 displays an illustration of this model.

**Figure 13:**
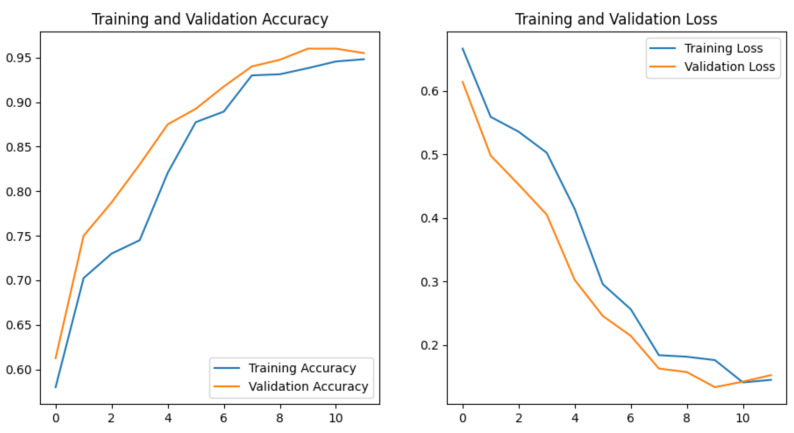
Values for accuracy and loss functions in the CNN training process.

In the feature extraction stage, this model has five 2D-convolutional layers with 16, 32, 64, 64, and 64 3 × 3-filters, respectively. After each convolutional layer, there is a 2 × 2 max pooling layer. After these stages, the output is flattened, and submitted to a dense layer with 1024 neurons. Up to this point, the convolutional and dense layers used a rectified linear unit (ReLU) activation function. Finally, the output is a single neuron with a sigmoid activation function. The loss function was also the cross-entropy, and we trained the function for 12 epochs, which was an empirical value found as the maximum epochs to avoid overfitting signs. We also tested this model according to the hardware and software performance indicators to answer the raised questions. Figure 13 displays the behavior of the loss and accuracy during the model training.

### 4.6. Validation Tests

After proposing the hardware, software, and architectural elements, we must also establish metrics to validate each stage. In this subsection, we display the modeling and metrics to evaluate each element. For the hardware elements, we evaluated the performance of the edge AI server within the various platforms. For the software traits, we examined the metrics of the ML software predictions for the proposed application. Finally, for the architecture, we examined the timing constraints for multiple clients connected to the edge AI server, considering a real-time QoS test.

#### 4.6.1. Hardware Validation Tests

There are two main hardware elements involved in this appliance: A smart-helmet and an edge AI server node. The helmet was previously created to support multipurpose applications in this area. Thus, the validation stage considers that validation is necessary considering the newly-added element: The edge AI node.

In Figure 6, we established the tasks performed both by the smart helmet and edge AI node. At first, we need to evaluate the hardware elements for each of the proposed solutions according to their respective distributor sites. The candidate solutions are the Raspberry Pi Zero W, Raspberry Pi 3B, Raspberry Pi 3B+, and Jetson Nano. Table 1 presents the most relevant aspects about each solution.

For this test, we perform the tasks described in Figure 6 pipeline for each candidate. We evaluate the latency to perform all internal stages for each solution, running the same code to receive the data from a client, predicting the result (healthy or diseased), returning, and storing the predictions in a text file. For the hardware evaluation, we consider only the latency in the stages performed locally. The networking-dependant parts will be later performed considering architectural traits. Finally, we also tested the average predictions per second ratio comparing the two software techniques. This provides the answer to one of the questions raised in the software proposal, regarding the model performance in the embedded hardware.

#### 4.6.2. Software Validation Tests

The novel proposed software is a ML-based prediction running in an edge AI server node. For this matter, we trained an MLP neural network model to predict if leaf images contain diseased or healthy leaves. We also tested the same metrics for the CNN model to verify the improvement on this trait using a more modern solution.

For completing software validation, we need to understand the performance of this ML model within the context of this data. Thus, we use traditional ML metrics to analyze the data. We evaluate the confusion matrix, as well as the *Precision*, *Recall*, and F1-Score metrics. The following equations display the formulae for these metrics. In these equations, TP is the number of true positives, FP is the number of false positives, TN is the number of true negatives, and FN is the number of false negatives:

(2) $$\begin{array}{c} Precision=\frac{TP}{TP+FP} \end{array}$$

(3) $$\begin{array}{c} Recall=\frac{TP}{TP+FN} \end{array}$$

(4) $$\begin{array}{c} F1-Score=2\times \frac{Precision\times Recall}{Precision+Recall}. \end{array}$$

#### 4.6.3. Architecture Validation Tests

We need to consider features that evaluate the proposed scenario’s individual and general performances for the architecture validation tests. Thus, we developed an experiment designed as a real-time QoS test based on similar studies concerning IoT and wireless sensor networks [49,50] to evaluate the real-time constraint. This test evaluates the capability of performing a set of tasks, considering both individual- and network-based conditions.

At first, we consider duration as discrete intervals, as the set $D=d_{i},i\in N$, where $d_{i+1}-d_{i}=\theta$, and θ is a constant sampling time. The soft real-time deadline will be represented by ϕ, where $\phi =k\times \theta ,k\in N^{*}$. Thereby, we establish the following definitions:

**Definition 1.**  *Let $D=d_{i}$ be the finite set of nodes performing IoT-dependant tasks, where i∈N;*

**Definition 2.**  *Let $E=e_{i}$ be the finite set of events that each node performs, where i∈N;*

**Definition 3.**  *Let $L=l_{g,e}$ be the length of time interval that the node g takes to perform an event e during the execution, where g∈G and e∈E;*

**Definition 4.**  *Let $P=p_{i}$ be the set of patterns of events to be observed in the devices, where $p_{i}=E_{i}$, $E_{i}\subset E$, and i∈N. In this case, all client devices will perform the same events in the same pattern.*

**Definition 5.**  *Let $O=o_{i}$ be the finite set of observations of a certain pattern $p_{i}\in P$ on each device;*

The equation that represents the elapsed time λ to observe a particular pattern $p_{i}\in P$ is:

(5) $$\lambda_{o_{i}}=\sum l_{g,e_{k}}|\forall e_{k}\in o_{i},o_{i}=O_{p_{i}}.$$

All client devices in the network composition will have the same ϕ soft real-time deadline. Given this equation, let $\hat{O}$ be a subset of *O*, where $\lambda_{o_{i}}\leq \phi$, $\forall o_{i}\in \hat{O}$. Finally, given the sets *O* and $\hat{O}$:

**Definition 6.**  *Let N be the number of elements on the set O;*

**Definition 7.**  *Let $N_{h}$ be the number of elements on the subset $\hat{O}$.*

The quality factor $Q_{f}$ will be represented by the following equation:

(6) $$Q_{f}=\frac{N_{h}}{N}\left(\times 100\%\right).$$

This result represents how often the nodes execute a pattern of events without violating the soft real-time constraints. The clients represent the smart-helmets and will send data to the edge AI server node in parallel on each test.

### 4.7. Case Study Validation for Deployment

To validate the system within the case study, we used a test based on the probability distribution function presented in Equation (1). This equation describes the probability of finding diseased leaves in a sampling process around the tree, based on the spatial coordinates. The maximum value of this function is described by the $P_{0}$ value, while the spacial disease “epicenter” is located on the $\left(x_{0},y_{0},z_{0}\right)$ coordinate. This condition is illustrated in Figure 2, presented in Section 3.

**Figure 14:**
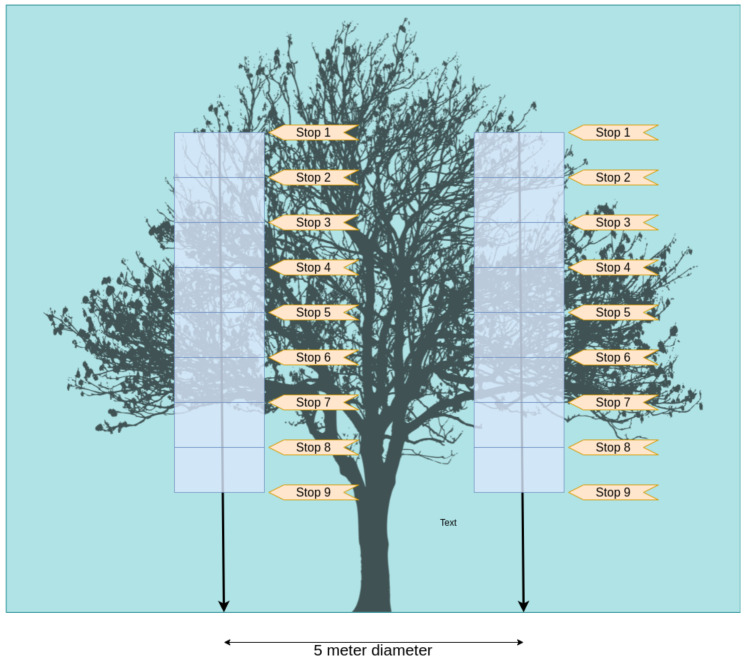
Sampling process illustration.

In this validation test, we consider a team of three climbers sampling leaves in individual heights within transects of the canopy. The location of the three climbers is arbitrary but known. The stops of the transect are also known, making it possible to map their location along the process as a three-dimensional point. The researchers should be located within an arbitrary diameter from a tree trunk center in the canopy for better spatial distribution. Figure 14 displays this organization for a 5-meter radius and 9 stops.

**Figure 15:**
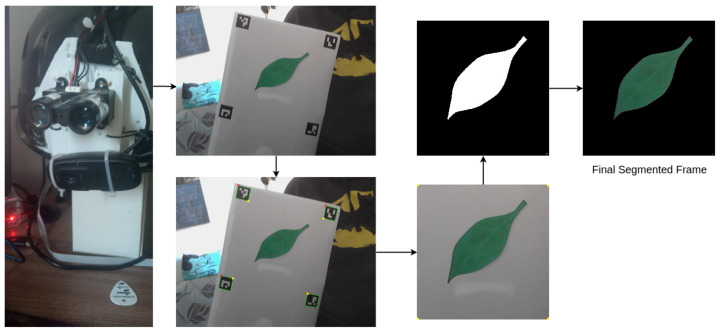
Demonstration of the segmentation process. The prototype used a USB camera to capture the data, which can be processed by the prototype itself or in the edge AI server node.

We considered a process with an arbitrary number of stops on the climb, according to the demand of the transect method. At each stop, the researcher would sample leaf images. The system automatically classifies the sampled leaves as healthy or diseased according to the obtained method. Thus, for each (x,y,z) coordinate where a researcher samples the leaves using the helmet and a background template, the system can calculate the percentage of diseased unities. As a climber can transport small objects to the canopy, we propose the usage of a background template, which is a solid object with identification tags on its borders. This proposal allows the algorithm to avoid background interference in the sampling process. We developed a prototype test to demonstrate this issue using the wearable camera. The prototype acquires the image, identifies some tags in the background template, performs a four-point transform to separate the region of interest, and uses Otsu’s binarization to segment the image. Figure 15 displays the pipeline used in this study.

With this information, the system performs a regression fit to the PDF described in Equation (1). For this matter, it must obtain the parameters within the tuple $T=(p_{0},\sigma ,x_{0},y_{0},z_{0})$. We chose to perform this task using an evolutionary algorithm, considering the tuple candidates as the genotype and the mean squared error as the fitness function. This choice was based on three main aspects:

- *Ease of use:* It is easier to perform regression for a smooth parametric arbitrary three-dimensional distribution function with an evolutionary algorithm than designing an interpolation based in various parameters and kernel functions;
- *Flexibility:* The same process can be used to obtain a regression to any parametric model by just changing the input parameters on the same algorithm;
- *Robustness:* The regression algorithm displayed robust results, even with a change on its parameters.

In this example, the climbers would have nine stops, sampling 200 leaves within each of them. The system automatically classifies each leaf, sending information about each location and the compressed leaf image. For simulating the sampling process, we sampled 100 random images from the original dataset. Our selection system generates a random number and compares it with the PDF on Equation (1) with arbitrary parameters. We chose the value of $p_{0}$ as 0.65, considering the maximum incidence of diseased leaves in the study of García-Guzman et al. [35]. We also considered the coordinates of the tree trunk and ground as (0,0,0) origin and arbitrarily selected (2,−2,8) as the disease epicenter. Finally, our σ value was 5. Thus, the final arbitrary PDF for this test is:

(7) $$P\left(x,y,z\right)=0.65.e^{-\frac{{\left(x-2\right)}^{2}+{\left(y+2\right)}^{2}+{\left(z-8\right)}^{2}}{10}}.$$

**Figure 16:**
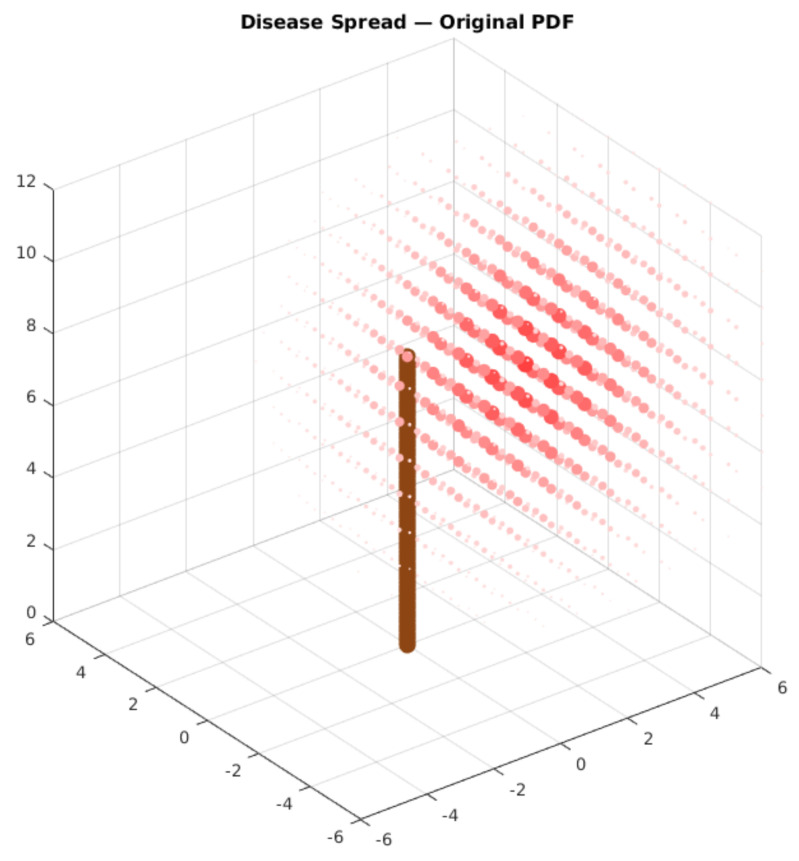
Arbitrary PDF display. The larger and more colorful red dots have a bigger probability density. The brown cylinder represents the main tree trunk.

Finally, the system classifies and stores the information about the image. We expect to perform the analysis with the stored data to obtain the original PDF values using an evolutionary algorithm. The edge AI node can also perform this analysis to provide in-field insights from the sampled data. The objective is to get as close as possible to the original values of Equation (7). Figure 16 displays the spatial distribution of the disease in this arbitrary function. The more colorful and bigger the red circle, the greater the probability of finding diseased leaves on this coordinate. The brown stick indicates the position of the main tree trunk.

## 5. Results

The previous section presented all the hardware, software, and architecture aspects applied in the proposed case study. Furthermore, we displayed the evaluation metrics used to validate each branch on the co-design reviewed pattern. Finally, we discussed the validations for an application within the case study. In this section, we display the results obtained from experiments considering the proposed elements.

### 5.1. Hardware Validation Tests

**Figure 17:**
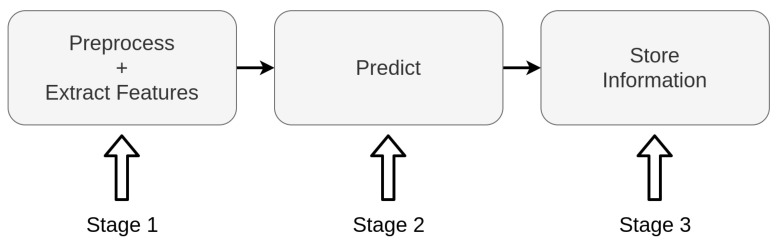
Pipeline for the hardware validation test.

In Section 4, we present the hardware specifications for the edge AI server node. All candidates are COTS computer-on-modules. For validating the hardware candidate, we tested the performance of the candidates in realizing the internal edge AI tasks. Figure 17 displays the pipeline for the proposed test. As displayed, the internal tasks for the edge AI server are divided into three stages: (i) Preprocessing and extracting the feature vector, (ii) predicting the leaf condition, and (iii) storing the prediction data.

We performed the tests in all the candidates presented in Table 1. For the Jetson Nano, we performed the tests both in the 5 W and 20 W modes. We performed the following pipeline in all 437 images from the test set. The hardware candidates and configurations will henceforth be named *Zero W* (Raspberry Pi Zero W), *3B* (Raspberry Pi 3B), *3B+* (Raspberry Pi 3B+), *Jetson 5W* (Jetson Nano running in 5 W mode), and *Jetson 20W* (Jetson Nano running in 20 W mode).

The *Zero W* was tested given it is the computer in the helmet prototype. *3B* and *3B+* were tested as they have smaller costs than a Jetson Nano, although the processor configurations are similar. With *Jetson 5W* we seek to compare the most expensive candidate with limited hardware capabilities, as the OS disables half the CPU cores to save power in this economic operation mode. Finally, we wanted to verify the difference between the most expensive hardware in the most potent operation mode and the other candidates’ performance.

**Figure 18:**
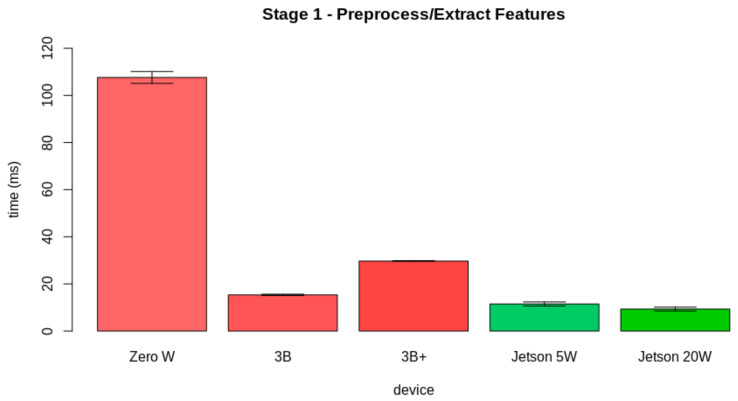
Latency results for the first stage.

At first, we evaluated the results for Stage 1. In this part, the hardware preprocesses the image, transforming its color space from RGB to HSV. Then, it extracts the pseudospectrum from the Hue channel. Figure 18 displays the results obtained from the evaluation of the latency from the first stage. *Zero W* took 107.61±2.53 ms to perform the first stage, *3B* took 15.34±0.28 ms to perform this task, *3B+* took 29.69±0.13 ms to perform this stage, *Jetson 5W* took 11.47±0.87 ms to perform this part, and *Jetson 20W* took 9.32±0.81 ms.

**Figure 19:**
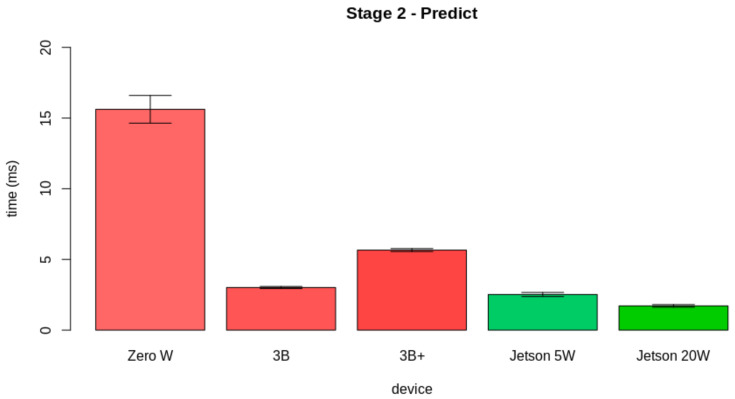
Latency results for the second stage.

Then, we assessed the results for Stage 2. This stage corresponds to the prediction of the leaf condition using the model. Figure 19 presents the results obtained from the evaluation of the latency from the second stage. *Zero W* took 15.62±0.98 ms to perform the first stage, *3B* took 3.01±0.07 ms to perform this task, *3B+* took 5.66±0.10 ms to perform this stage, *Jetson 5W* took 2.52±0.14 ms to perform this part, and *Jetson 20W* took 1.71±0.09 ms.

**Figure 20:**
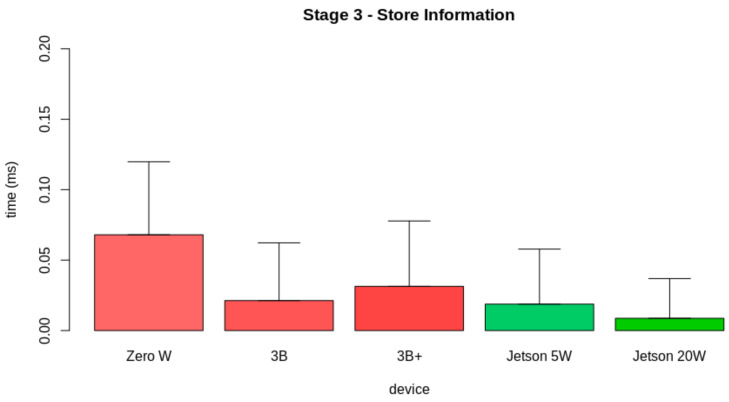
Latency results for the third stage.

Finally, we studied the results for Stage 3. This stage corresponds to the storage of the information obtained from the previous stages. Figure 20 shows the results obtained from the evaluation of the latency from the second stage. *Zero W* took 0.07±0.05 ms to perform the first stage, *3B* took 0.02±0.04 ms to perform this task, *3B+* took 0.03±0.05 ms to perform this stage, *Jetson 5W* took 0.02±0.04 ms to perform this part, and *Jetson 20W* took 0.01±0.03 ms. The apparent inconsistencies, in this case, happened as in many cases, the time interval of this stage was lower than the minimum stored value, causing many measurements to be performed in zero time.

**Figure 21:**
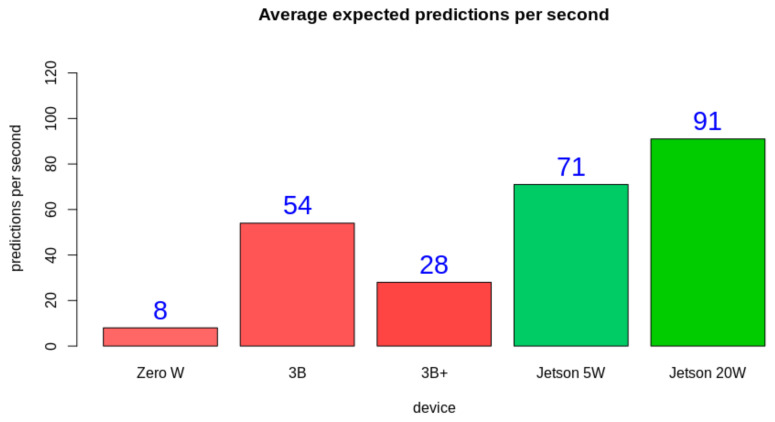
Average expected predictions per second ratio on each platform. The number in blue displays the expected ratio.

All the proposed hardware could perform the desired task. Thus, the choice comes from evaluating the performance, which could be compared later with the project cost. From the test results, it is possible to observe that even in the power saving mode, the Jetson Nano performed better than the Raspberry Pi 3B and 3B+. The Raspberry Pi Zero W has the lowest hardware power, and thus the worst performance. Even though the hardware specifications are very similar, the Raspberry Pi 3B performed better than the Raspberry Pi 3B+ and had a closer performance to the Jetson Nano operating in the 5 W mode. Figure 21 displays the average expected prediction per second rate for each platform.

As expected, *Zero W* had a very low performance. This justifies the initial architectural proposal involving another hardware element to bear the heavier processing tasks. Another expected result was that the highest performance was reached with *Jetson 20W*. An initial surprising result is that *3B* had a very superior result when compared to *3B+*, even though it has a better processor theoretically. Another important result is that even with two of the four cores disabled, *Jetson 5W* had a superior performance when compared to *3B* and *3B+* operating with four cores and more than two times the input power. This result makes *Jetson 5W* a great candidate for field processing, as it can operate at a high performance with such a power constraint. From these results, we consider that *3B* and *Jetson 5W* are the main candidates for bearing this application, as they have the best tradeoff between performance and power draw.

**Figure 22:**
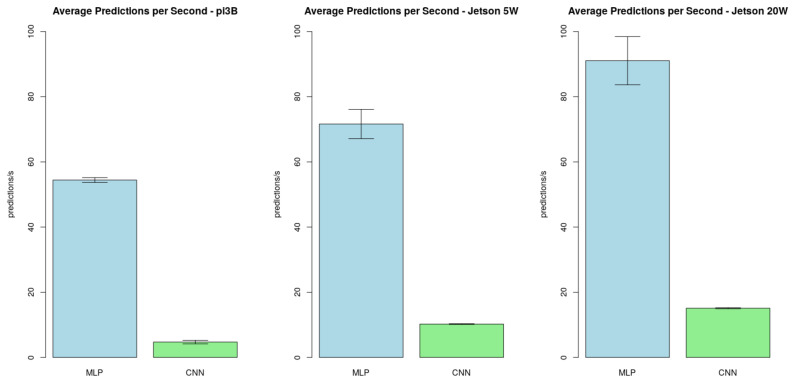
MLP and CNN performance comparison test results.

Finally, we tested the average predictions per second ratio running the CNN and the MLP pipelines. The only difference in the CNN pipeline from Figure 17 is that the CNN does not require a feature extraction process. Thus, this stage represents only the input manipulation to feed the model. We performed these tests in the main hardware candidates (*Jetson 5W*, *Jetson 20W*, and *3B*). The obtained results for the predictions per second in the proposed configurations were:

- The average predictions per second ratio in *3B* was 54±1 for the MLP pipeline and 5±0 for the CNN pipeline;
- The average predictions per second ratio in *Jetson 5W* was 71±5 for the MLP pipeline and 10±0 for the CNN pipeline.
- The average predictions per second ratio in *Jetson 20W* was 91±7 for the MLP pipeline and 15±0 for the CNN pipeline;

Figure 22 also displays these results in the respective cited order. This data indicates that even in case of improvements on the software results, the CNN model is not adequate for time-restrictive tasks in the proposed configurations. This model is suitable to perform a later review of in-field captured results, but not to be integrated into a distributed constrained environment within the context of these tests.

### 5.2. Software Validation Tests

As presented in the previous section, the software validation tests consider the traditional ML metrics. In this context, we evaluated *Precision*, *Recall*, and *F1-Score* metrics.

For simplicity matters, the validation set is sampled once from the training data. It has 10% of all images from the training data. Table 2 displays the obtained results for the validation set. The results display that the system could identify the diseased leaves in 90% of the cases. The Precision and Recall are also balanced, resulting in a balanced F1-Score. This result indicates that the amount of false positives and negatives is about the same. Table 3 displays the confusion matrix obtained from this stage.

For the test set, we also calculated the global average and the same traditional metrics. The test set was previously separated using 10% from the original dataset images. Table 4 displays the obtained results for the validation set and Table 5 displays the confusion matrix for this stage. Again, the results display that the system could identify the diseased leaves in circa 90% of the cases. Even with a small difference, the Precision and Recall are balanced, resulting in a balanced *F*1-*Score*. This result enforces the feasibility of the proposed algorithm within the proposed context.

From these stages, we conclude that the algorithm is valid for the proposed end. It identifies diseased and healthy leaves with circa 90% of accuracy, and the results are balanced. The following tests must validate architectural aspects from this solution. We also performed the same predictions considering the CNN. For this matter, we used the same test set to obtain the prediction results. Table 6 and Table 7 display these results.

As expected, the CNN performed better than the MLP. The results display a 5% improvement on the precision of the results. This result enforce the usage of this perspective in further investigations rather than the MLP model. In the perspective of using more computational power to analyze the data, the CNN is a preferable model than the MLP.

### 5.3. Architecture Validation Tests

**Figure 23:**
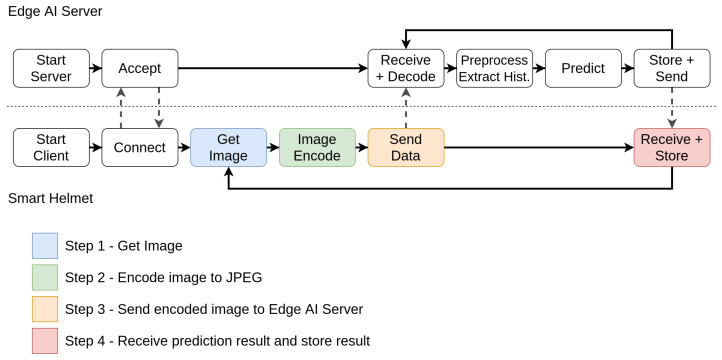
Stages considered in the architectural validation test.

The architecture validation test evaluates the capability of performing a task within a soft real-time constraint. It means it is a performance evaluation that provides an overview of the scalability of the proposed architecture. For this matter, we used the Jetson Nano as an edge AI server to perform the pipeline presented in Figure 6. For the client, we developed a version of this system that provides the latency information regarding the steps highlighted in Figure 23.

In this study, each step corresponds to an event on the modeling presented in Section 4.6.3. All clients can perform only the same set of events. The number of nodes performing the tasks corresponds to the number of devices performing the IoT-dependant tasks. In this test, we increase the number of clients and evaluate how this affects the soft real-time constraint.

**Figure 24:**
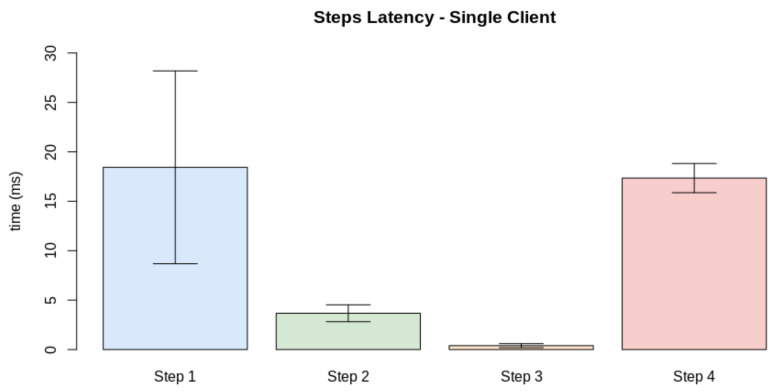
Latency for each of the steps presented in Figure 23.

Thus, we must at first evaluate the real-time criteria considering the pipeline presented in Figure 23. Thus, we performed the test considering the latency of the processes for a single client. The test also considers a number of finite discrete time blocks. In this context, we considered the minimum time block as 1 ms. Figure 24 displays the latency for each step in a single-client test. To define the soft real-time constraint (ϕ), we evaluated the minimum number of blocks necessary to provide the service for a single client with 100% quality (Qf=1.0), with an additional 10% margin for relaxing the criteria. Through this method, we defined ϕ=90 ms.

**Figure 25:**
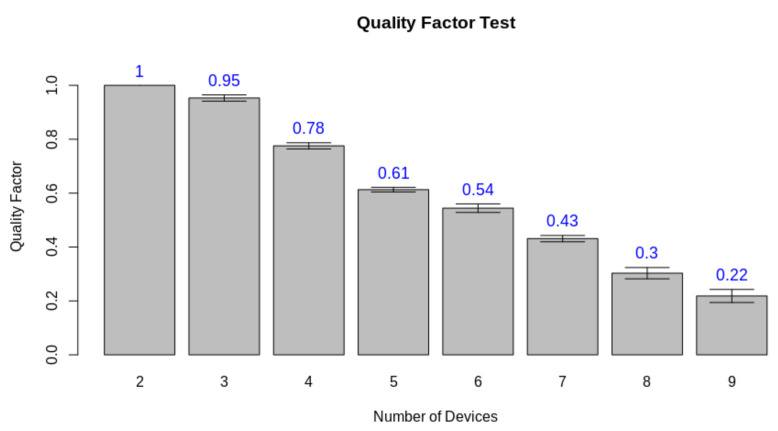
Quality factor test results.

After defining ϕ, we repeated the test instantiating 2 to 9 clients performing the same task. The instances were simulated in a computer machine connected to the WLAN network as the edge server. In each test, each instance performed the same test mentioned in Figure 23, measuring the time involved in performing each desired event. In the end, we measured the quality factor average and standard deviation, considering all nodes involved. Figure 25 displays the result for this test. This result displays that the quality of the edge AI-based service drops when the number of clients rises, considering a defined real-time constraint. Nevertheless, the system maintains high quality with a low number of connected clients.

**Figure 26:**
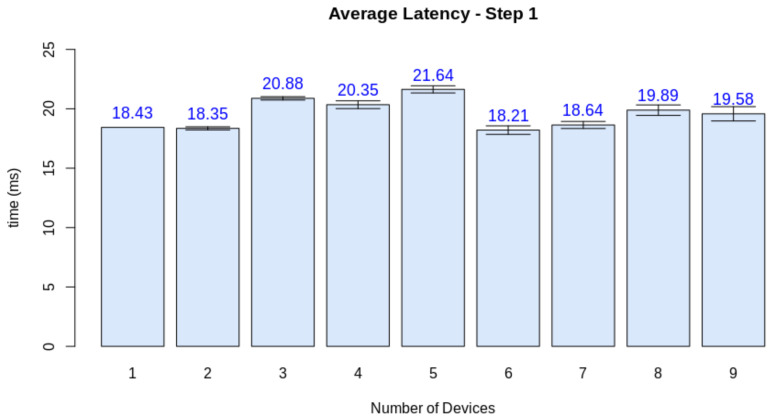
Latency test results for step 1.

**Figure 27:**
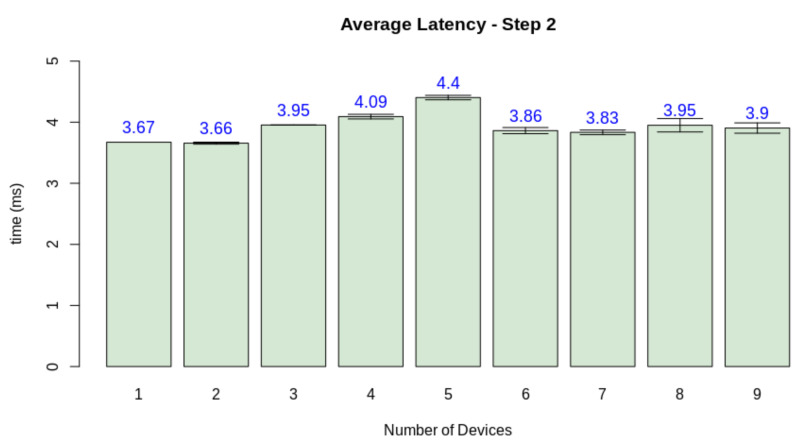
Latency test results for step 2.

**Figure 28:**
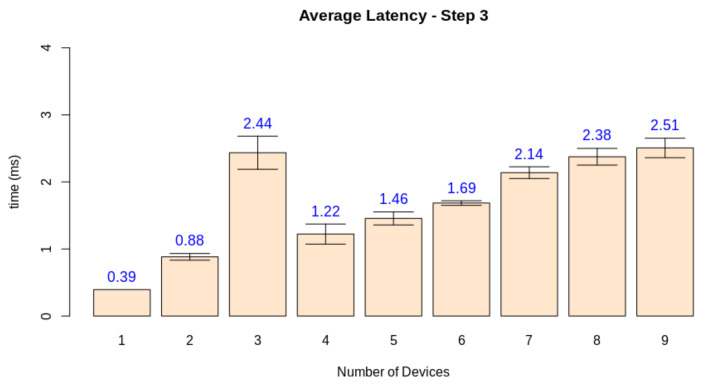
Latency test results for step 3.

**Figure 29:**
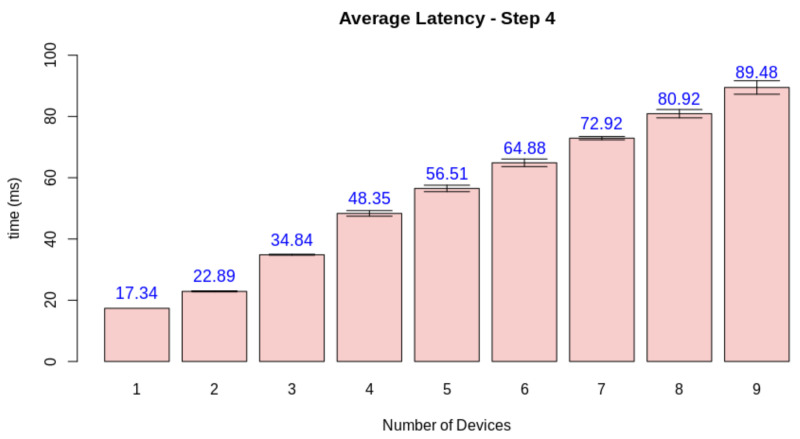
Latency test results for step 4.

Finally, we need to check if the quality loss happened due to the server overload or if other factors influenced the simulation software. For this matter, we measured the average latency of each stage when increasing the number of nodes. While steps one and two are device-dependant, step three can be jeopardized by a network overload, and step four depends on the performance of the edge AI node. Figure 26, Figure 27, Figure 28 and Figure 29 display the results for this analysis.

As expected, the latency in the first two steps did not suffer from the increasing number of clients. These stages are only dependant on the client performing its tasks. Step three presents the first network-dependent action. The increasing number of clients could cause a problem in the communication process. The results display that this overload increases the latency for this step, but the impact on the final result is minimal (circa 2 ms). Finally, stage 4 displays that this overload on the edge AI node is the main factor causing a decrease in quality given the increasing number of clients. This stage is both related to networking and the machine learning inference process.

### 5.4. Case Study Validation for Deployment

**Figure 30:**
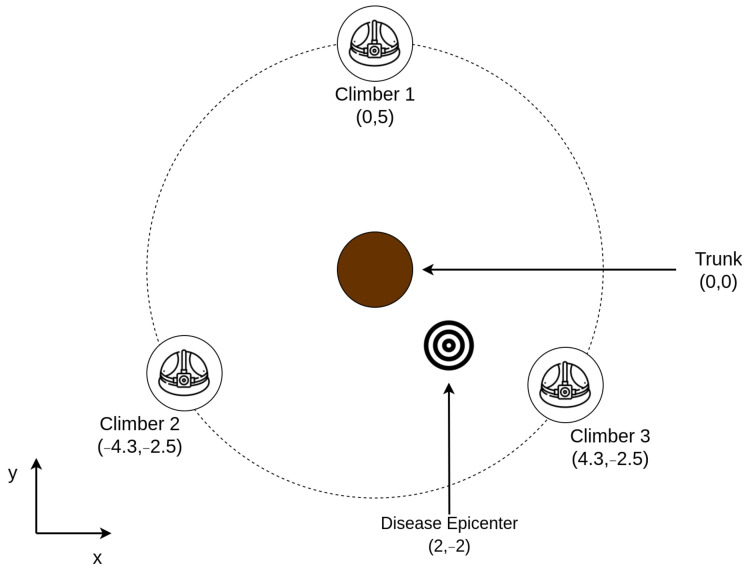
Upper view of the case study organization.

As reported in Section 4, we also performed a validation stage for the complete solution. For this matter, we developed a simulation from a case study appliance for the proposed solution. In this application, three researchers sample 200 random leaves in specific heights, performing the cylinder method. The edge AI server node predicts the state for each leaf and stores it together with the researcher coordinate. In this appliance, the researchers are located in a 5-meter diameter circle around a tree trunk. Figure 30 displays how these devices are organized around the tree trunk.

We used Equation (7) as the baseline for randomly selecting leaves from the diseased and healthy sets. First, the probability baseline was calculated based on the (x,y,z) coordinate of each researcher on the presented point. Then, for each of the 200 samples, the program generates a random number in the [0,1) interval. If this value is lower than the baseline probability, the algorithm selects a diseased leaf. Otherwise, it selects a healthy one.

**Figure 31:**
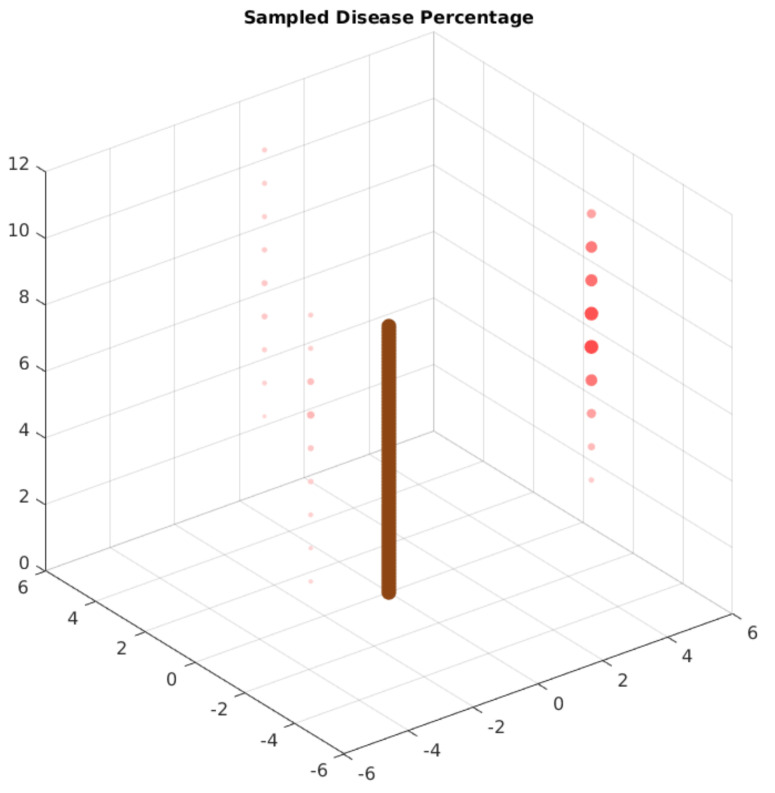
Case study sampling distribution. The larger and more colorful red dots have a bigger percentage of diseased leaves. The brown cylinder represents the main tree trunk.

After this process, we performed a test with the trained model. For each device and location, the test application predicts the leaf conditions from each sample. With this data, the application calculates the percentage of diseased leaves, generating a distribution sample. Figure 31 displays the results of the sampling process, considering the organization displayed in Figure 30. As in Figure 16, the larger and more colorful the red points, the higher the prevalence of diseased leaves.

Finally, we used an evolutionary algorithm to perform a regression to the parametric PDF presented in Equation (1) using the sampled data. Some features of this algorithm are:

- Each individual genotype is a $T=(p_{0},\sigma ,x_{0},y_{0},z_{0})$ tuple;
- The population has 100 individuals;
- Each round generates 70 offspring (30% elitism);
- Each round has a complementary local search in half the population;
- The algorithm stops with a convergence criteria and RMSE lower than 0.05 (5%).

To understand the level of prediction it is, we ran the model 20 times, and evaluated the average value for each paramenter of the $T=(p_{0},\sigma ,x_{0},y_{0},z_{0})$ obtained from the best individual of the population. The average responses obtained from this experiment are:

- $p_{0}=0.65\pm 0.03$. The original value was 0.65;
- σ=12±0.86. The original value was 5;
- $x_{0}=1.96\pm 0.21$. The original value was 2;
- $y_{0}=-1.52\pm 0.35$. The original value was −2;
- $z_{0}=8.1\pm 0.16$. The original value was 8.

**Figure 32:**
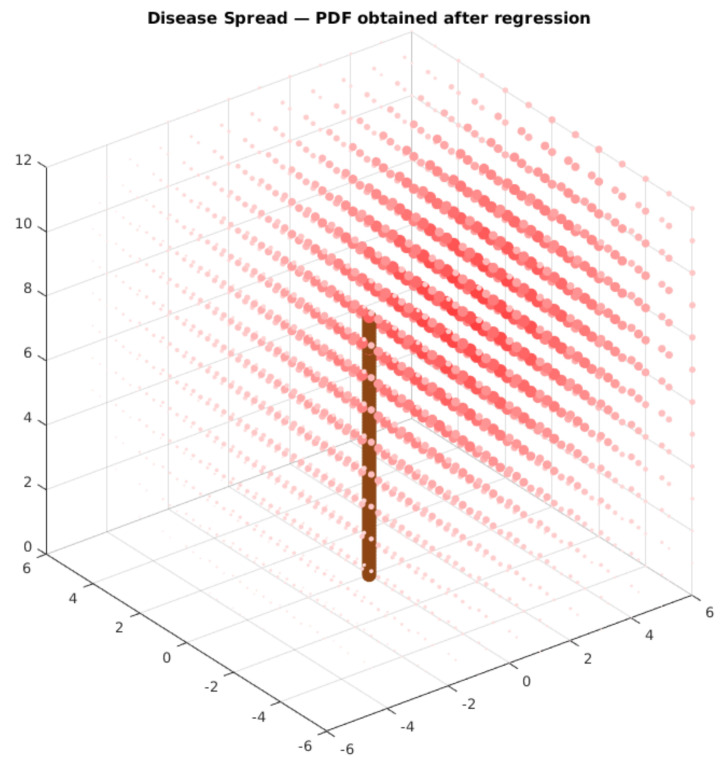
Estimated PDF display. The larger and more colorful red dots have a bigger probability density. The brown cylinder represents the main tree trunk.

Figure 32 displays the estimated spatial distribution of the disease according to these parameters. The obtained values are very similar to the expected results. The distance from the estimated epicenter of the disease and the original PDF is 0.48 m. The value of the maximum estimated percentage is very similar to the original one. The dispersion of the estimated model is larger than the one in the original module. This difference may be caused by the uncertainty of the leaf classification model (circa 10%). Even with this uncertainty, the model provided a good estimation for the disease spread parameters, given the sampled data. We tested varying the algorithm’s parameters to verify if the obtained results would change. Our results displayed that increasing or decreasing the population size, the number of offspring, and maximum epochs had a minor impact on the obtained results. This result enforces that the process is very robust to obtain the model parameters.

## 6. Conclusions

Wearable edge AI is a perspective that enables the creation of several applications using various devices. Thus, this paper discussed the main aspects of creating wearable edge AI applications, especially for field research. Initially, we reviewed the HW/SW co-design pattern to enforce the parallel architectural design and validation. Finally, we performed an extensive case study to validate this process towards designing novel applications for ecological environment research.

### 6.1. A Novel Co-Design Approach

Traditionally, the hardware/software co-design principles consider the parallel development of hardware and software, with the integration after validating both traits. Here, we proposed a novel approach, considering that architectural decisions affect the hardware and software effects. Thus, we understand that it is necessary to validate the architecture integration among the software and hardware in our approach.

The proposal of a novel co-design approach starts similarly, with the requirements definition and general architecture proposal. Then, it splits into three branches. The architectural branch must identify how the devices communicate within the network, develop and integrate into parallel with HW/SW elements, and have its validation routines. The software and hardware branches are the same as the traditional co-design approach.

As the integration is parallel to the development of hardware and software modules, the last stage is to deploy the model in a validation case study. Thus, we proposed an appliance based on methods developed by ecologists to study forest canopies.

### 6.2. Developing a Wearable Edge AI Appliance

Our case study targets the usage of wearable edge AI in the forest environment. This kind of environment has an important edge requirement, as it has a complex connection and resource restraints. Thus, the appliance must be cloud-independent and provide a machine learning inference process for several devices in an IoT environment. Furthermore, we targeted this study to identify leaf diseases, as it has consequences and provides insight into ecosystem health.

We started from multipurpose wearable solutions, where the proposed hardware was previously validated. We then provided validation tests for the edge AI server node hardware. Furthermore, we validated an ML-based leaf classification as our edge AI software. We trained a software tool starting from an existing dataset and validated it according to the usual metrics from the area. We tested the quality of the provided edge AI service for the architecture validation, considering it a soft real-time application. We verified the quality loss for each added client.

Our results indicate the feasibility of this system. Usual COTS computer-on-modules were able to provide the required services in a prototype environment. Our result indicates that the modern computer-on-modules provide more resources to decrease the latency, even for CPU-constrained tasks. With this feature, these tools were able to perform better than older hardware with similar CPU chips. The software is a machine-learning- and computer-vision-based classifier. It was able to classify diseased leaves with 90% accuracy. The application was able to bear a few clients with a less significant quality-of-service loss.

The perspective of using deep learning to perform this task was also tested in this context. Although this process improves the accuracy to 96%, it was not suitable for time-sensitive insights in this configuration. Nonetheless, this process is indicated to review and improve the results of data obtained in field when reviewing this process results after the study. Further future works should also investigate how to enhance the CNN performance using specialized embedded hardware.

We then validated the proposed architecture within the case study. We randomly sampled leaves according to an arbitrary probability density function in three distributed space locations. We then used the model to classify the given leaves and used an evolutionary algorithm to perform regression for our PDF parameters. With the model, we were able to detect four of the five parameters of the PDF with a good approach. This result provided the position of the disease epicenter with a 50-cm precision in a 6 m × 6 m × 12 m space. It also was able to determine the maximum probability accurately.

### 6.3. Final Considerations

These results enforce our review proposal on the co-design process, considering the architectural integration as a parallel branch with the hardware and software. Our results also indicate the feasibility of the proposed architecture within the context of the case study. We were also able to use validated wearable tools to perform a case study considering all proposed elements. Future work should put these tools to proof in the actual field context, providing real-time insights for researchers. Future perspectives should also validate this novel co-design approach within various areas, such as industry and healthcare, for instance.

## Figures and Tables

**Table 1:** Hardware specifications for the edge AI server node candidates.

	Raspberry Pi Zero W	Raspberry Pi 3B	Raspberry Pi 3B+	Nvidia Jetson Nano
**CPU**	1× ARM11 @ 1 GHz	4× ARM Cortex-A53 @ 1.2 GHz	4× ARM Cortex-A53 @ 1.4 GHz	4× ARM Cortex-A57 @ 1.43 GHz
**RAM**	512 MB	1 GB	1 GB	4 GB
**Storage**	MicroSD card	MicroSD card	MicroSD card	MicroSD card
**Nominal** **Power**	5 V over microUSB (max. 6 W)	5 V over microUSB (max. 12.5 W)	5 V over microUSB (max. 12.5 W)	5 V over P4 Jack Barrell (max. 5 W/20 W modes)
**Network** **Platform**	2.4 GHz 802.11n	2.4 GHz 802.11n	2.4 GHz/5 GHz 802.11b/g/n/ac	2.4 GHz 802.11n (over USB)

**Table 2:** Metric results for the validation dataset. This set was obtained separating 10% of the training data for validation.

Global Accuracy: 90%				
	**Precision**	**Recall**	**F1-Score**	**Support**
healthy	0.89	0.90	0.90	198
diseased	0.90	0.90	0.90	209

**Table 3:** Confusion matrix for the validation data.

	Healthy	Diseased
**Healthy**	178	20
**Diseased**	21	188

**Table 4:** Metric results for the test dataset. This set previously separated, taking 10% of all images.

Global Accuracy: 91%				
	**Precision**	**Recall**	**F1-Score**	**Support**
healthy	0.93	0.88	0.91	217
diseased	0.89	0.93	0.91	220

**Table 5:** Confusion matrix for the test data.

	Healthy	Diseased
**Healthy**	192	25
**Diseased**	15	205

**Table 6:** Metric results for the test dataset—CNN results. This set is the same previously separated for the MLP.

Global Accuracy: 96%				
	**Precision**	**Recall**	**F1-Score**	**Support**
Healthy	0.96	0.95	0.96	217
Diseased	0.95	0.96	0.96	220

**Table 7:** Confusion Matrix for the test data—CNN results.

	Healthy	Diseased
**Healthy**	207	10
**Diseased**	9	211

## Abbreviations

The following abbreviations are used in this manuscript:

AI	Artificial Intelligence
COTS	Commercial-Off-The-Shelf
CV	Computer Vision
HW	Hardware
IoT	Internet of Things
ML	Machine Learning
NLP	Natural Language Processing
SW	Software
WBAN	Wireless Body Area Network
WLAN	Wireless Local Area Network
WPAN	Wireless Personal Area Network
